# Assessment of Vitamin E and Glutathione Peroxidase Levels in Salivary Samples of Children With and Without Dental Caries in Erbil City, Iraq

**DOI:** 10.7759/cureus.74872

**Published:** 2024-11-30

**Authors:** Pareehan M Hussein, Vian M Hussein

**Affiliations:** 1 Dental Surgery, Hawler Center, Erbil, IRQ; 2 Dental Public Health, Kurdistan Higher Council of Medical Specialties, Erbil, IRQ

**Keywords:** childeren, dental caries, glutathione peroxidase, salivary samples, vitamin e

## Abstract

Introduction: According to the classic understanding of the etiology of dental caries, an imbalance between demineralization and remineralization in the oral cavity is important. Salivary antioxidants, including glutathione peroxidase and vitamin E, can modulate oxidative stress.

Methods: This cross-sectional study evaluated the levels of glutathione peroxidase and vitamin E in the saliva of 90 children from Erbil City. The children were further subdivided based on their caries status and membership in the caries-free group. SPSS software was used for statistical comparisons.

Results: The difference in antioxidant levels between the groups was statistically significant. In children, a significant decrease in the levels of glutathione (1664.356 µM) and vitamin E (4.0844 nM) in caries-susceptible individuals was observed compared to those not affected by caries (glutathione, 1945.355 µM; vitamin E, 5.3240 nM).

Conclusion: The study postulates that lower levels of antioxidants are associated with dental caries and highlights the potential of glutathione and vitamin E for oral health protection. This calls for further exploration of the usefulness of these antioxidants as potential therapeutic mediators for dental care.

## Introduction

Dental caries, commonly known as tooth decay, remains a significant public health challenge globally, particularly among children. Despite advancements in dental care, it continues to be one of the most prevalent chronic diseases, affecting children’s overall health and well-being [1]. The development of dental caries is multifactorial, involving complex interactions among microbial activity, dietary habits, individual susceptibility, and time. The process begins with the demineralization of tooth enamel. This occurs when acids, produced by bacteria during the fermentation of dietary carbohydrates, erode the mineral content of enamel, ultimately forming cavities. Various protective mechanisms, including those provided by saliva, play a pivotal role in preventing and controlling this disease [1].

Saliva serves as a crucial component in maintaining oral health by acting as a buffer against harmful acids and a medium for the delivery of essential minerals such as calcium, phosphate, and fluoride. These minerals are vital for the remineralization of tooth enamel, counteracting the effects of demineralization. Additionally, saliva contains a wide range of antimicrobial proteins and enzymes that regulate microbial populations, thereby safeguarding oral tissues [2,3]. Imbalances in the delicate equilibrium between demineralization and remineralization can predispose individuals to dental caries [4].

Among the bioactive components of saliva, antioxidants such as vitamin E and glutathione peroxidase (GPx) have garnered considerable attention due to their potential protective roles [5]. Oxidative stress, defined as an imbalance between reactive oxygen species (ROS) and the body's antioxidant defenses, is implicated in numerous diseases, including dental caries. While ROS serve necessary physiological roles in small amounts, their accumulation can cause tissue damage, including in the oral cavity [4,6].

Vitamin E, a fat-soluble antioxidant, protects cell membranes from oxidative damage by neutralizing free radicals. In the context of oral health, its presence in saliva may help mitigate the oxidative damage caused by bacterial activity, thereby reducing enamel breakdown. Similarly, GPx is a potent enzymatic antioxidant that detoxifies harmful by-products, such as hydrogen peroxide. By maintaining oxidative balance, GPx can protect oral tissues and potentially reduce the risk of dental caries [7,8].

Research into the protective roles of salivary antioxidants has expanded in recent years. While some narrative reviews have summarized the potential of antioxidants in oral health, original studies are needed to elucidate these relationships. For example, antioxidant properties of saliva have been explored in various studies, but evidence directly linking antioxidant levels to caries prevention remains limited. Investigating these relationships could provide new perspectives on caries prevention [9,10].

Antimicrobial properties of saliva also contribute significantly to oral health. Proteins such as lactoperoxidase and peptides such as histatins and defensins help control bacterial growth in the oral cavity. These components act synergistically to maintain a balanced microbial ecosystem, preventing conditions conducive to caries formation [11,12].

This study, conducted in Erbil City, Iraq, focuses on examining the levels of vitamin E and GPx in the saliva of children with and without dental caries. By comparing these groups, the research aims to identify the potential protective roles of these antioxidants in reducing caries susceptibility. While previous studies have highlighted the importance of salivary components in oral health, this study seeks to provide a clearer understanding of how specific antioxidants contribute to caries prevention [13,14].

By clarifying the antioxidant properties of saliva and their relevance to dental health, this research could inform future strategies for caries prevention. These may include dietary recommendations to enhance antioxidant intake or the development of dental products enriched with antioxidants to strengthen natural oral defense mechanisms [15-19]. Ultimately, this study aims to contribute to reducing the global burden of dental caries, improving children’s oral health outcomes, and enhancing their quality of life.

## Materials and methods

Study design and setting

This cross-sectional study aimed to assess vitamin E and GPx levels in salivary samples of children with varying dental caries status. A total of 90 children, aged 6-12 years, were recruited between January 15, 2023, and February 11, 2024. The study was conducted at three major dental centers in Erbil City, Iraq: Azadi, Hawler, and Khanzad. The participants were categorized into two groups: those with dental caries (caries-active group) and those considered caries-free based on their dental health status.

Sample size and sampling technique

The sample size was determined based on convenience sampling, targeting patients who visited the designated centers during the study period and met the inclusion criteria. All children presenting with dental issues at the centers were screened, and those who fit the eligibility criteria were invited to participate. This approach ensured accessibility and feasibility for obtaining the desired sample size.

Eligibility criteria

Inclusion Criteria

Children aged 6-12 years with a minimum of five decayed, missing, or filled teeth (DMFT ≥5) were classified as the caries-active group, while those with a DMFT score of <5 were considered caries-free. Only children whose parents or caregivers provided informed written consent were included.

Exclusion Criteria

Children younger than six or older than 12, those with systemic illnesses, salivary disorders, or white spot lesions, and children whose parents refused participation were excluded.

Data collection and assessment

Data collection involved a structured, validated questionnaire [20], developed in English and translated into the local language for ease of comprehension. The questionnaire consisted of:

Dental Health Assessment

Using the DMFT caries index, the number of DMFT was documented. Although DMFS (decayed, missing, and filled surfaces) is recognized for higher sensitivity, DMFT was selected for its ease of implementation in pediatric populations. Future studies may incorporate DMFS for enhanced accuracy.

Sociodemographic Information

Data on age, gender, parental education, and socioeconomic status were collected.

Salivary Biomarkers

Saliva samples were collected under standardized conditions (unstimulated samples, 9-11 AM) to minimize variability. Each participant sat upright with a slight tilt of the head and expectorated saliva into pre-labeled polypropylene tubes every 30 seconds over a period of five minutes. Levels of vitamin E (µmol/L) and GPx (µmol/L) were subsequently analyzed using biochemical assays.

Calibration of examiners

To ensure data accuracy, dental health assessments were performed by trained and calibrated examiners. The calibration process involved assessing inter- and intra-examiner reliability through kappa statistics (≥0.80), reflecting high consistency. This step reduced measurement bias and enhanced the reliability of the DMFT scoring.

Questionnaire validation

The questionnaire underwent rigorous validation, including content and construct validation by a panel of experts in pediatric dentistry and public health. A pilot test with 10 participants was conducted to evaluate clarity, relevance, and reliability (Cronbach’s alpha: 0.85). Adjustments were made based on feedback before implementation.

Ethical considerations

This study received ethical approval from the Dental Research Ethics Committee of the Kurdistan Higher Council of Medical Specialties (Approval Code: DREC/2023-01). Informed written consent was obtained from parents or caregivers of all participating children, ensuring adherence to ethical research practices.

Data management and statistical analysis

Data were recorded using a specially designed electronic database. Statistical analyses were performed using IBM SPSS Statistics for Windows, Version 28.0 (Released 2021; IBM Corp., Armonk, New York, United States). Descriptive statistics included rates, ratios, frequencies, and percentages. For inferential analysis, an independent t-test was used to compare mean salivary vitamin E and GPx levels between caries-active and caries-free groups, and a chi-square test was applied to evaluate associations between categorical variables, such as sociodemographic characteristics and caries status. Results were considered statistically significant at p ≤ 0.05.

Confounding factors

Potential confounders, such as dietary habits, oral hygiene practices, fluoride exposure, and parental education, were documented. These factors were controlled in the analysis to enhance the study’s validity. However, limitations in completely accounting for these confounders were acknowledged.

Grouping criteria and interpretations

Participants with no caries (DMFT = 0) were classified as caries-free, while those with DMFT scores between 1 and 4 were considered as having low caries. Variations in biomarker levels within these subgroups, especially among caries-free participants, were carefully interpreted to ensure meaningful conclusions.

## Results

A total of 90 participants were enrolled in this study. They were divided into two groups: caries and normal. The sex distribution showed that 50 (55.6%) of the participants were female and 40 (44.4%) were male (Table 1).

**Table 1:** General characteristics of participants.

Variable	Categories	Frequency	Percent
Study group	Caries-active	45	50
	Caries-free	45	50
Sex	Male	40	44.4
	Female	50	55.6
Total		90	100

The mean age of participants was 8.50 ± 1.247 years. Regarding antioxidant biomarkers, the overall mean level of glutathione was 1804.85 ± 516.28 μM, and the mean level of vitamin E was 4.70 ± 2.36 nM. Details of these measures, along with their respective ranges, are summarized in Table 2.

**Table 2:** Mean age, glutathione, and vitamin E of respondents.

Variable	N	Range	Minimum	Maximum	Mean	Standard deviation
Age (years)	90	6	6	12	8.50	1.247
Glutathione (μM)	90	2592	19	2611	1804.85	516.28
Vitamin E (nM)	90	14.20	0.60	14.80	4.70	2.36

Table 3 presents a comparative analysis of glutathione and vitamin E levels between the caries-active and caries-free groups. For glutathione, the caries-active group had a mean level of 1664.36 ± 561.02 μM, while the caries-free group exhibited a significantly higher mean level of 1945.36 ± 429.00 μM (t = -2.685, p = 0.009). Similarly, the mean level of vitamin E in the caries-active group was 4.08 ± 1.88 nM, which was significantly lower than the caries-free group's mean of 5.32 ± 2.65 nM (t = -2.589, p = 0.012).

**Table 3:** Comparative analysis of glutathione and vitamin E levels between caries-active and caries-free groups.

Variable	Study Group	N	Mean	Standard deviation	t-value	p-value	Significance
Glutathione (μM)	Caries-active	45	1664.36	561.02	-2.685	0.009	Significant
	Caries-free	45	1945.36	429.00			
Vitamin E (nM)	Caries-active	45	4.08	1.88	-2.589	0.012	Significant
	Caries-free	45	5.32	2.65			

## Discussion

Among oral diseases, dental caries is the most common disease affecting humans and wildly alters the respective salivary parameters [21]. This study aimed to explore the association between oxidative stress markers, specifically glutathione and vitamin E levels, and the presence of dental caries in children aged 8-12. Participants were divided into two groups: Group A (with dental caries) and Group B (without dental caries). The results demonstrated a notable decrease in glutathione and vitamin E levels among children with caries compared to their counterparts without caries. This finding underscores the role of oxidative stress in the pathophysiology of dental caries, although the limitations of the study design warrant cautious interpretation. These markers are essential for understanding the oxidative mechanisms underlying caries development and identifying potential therapeutic targets [22,23]. Another study examined vitamin E and GPx levels in children with and without dental caries. Children with caries had significantly lower levels of these antioxidants, suggesting a compromised oxidative defense mechanism in these individuals [24]. Another study supports the hypothesis that oxidative stress plays a crucial role in the pathophysiology of dental caries and that antioxidant supplementation could be a viable preventive strategy [25].

The observed decrease in glutathione levels aligns with prior research emphasizing oxidative imbalances in individuals with dental caries. A notable study by Pyati et al. [26] reported a correlation between the redox values of reduced glutathione and increased oxidative stress in persons affected by caries, thereby proposing potential compromise values in the oxidative defense mechanism. Nonetheless, the findings of this study contrast with those reported by Lizzo et al. [27], who found no difference among the respective groups. The differences between these two studies are attributed to population genetics, environmental reasons, and all other possible methodological issues, such as sample size and sample collection, which differ across these two studies. Similarly, there was a significant decrease in vitamin E levels among patients with caries compared to patients without caries. These results agree with those of Rios et al. [28], who reported that patients with caries had a significantly lower level of vitamin E. However, vitamin E has many important roles in upholding oral health.

Conversely, according to a study by Karthika et al. [22], sex differences were not observed in the levels of antioxidants within cohorts, likely due to different nutritional conditions or demographic factors. Overall, the marked difference in the antioxidant levels between the caries and non-caries groups may further exemplify the importance of antioxidants in the pathophysiology of dental caries. These differences highlight the multifaceted nature of oxidative stress in caries pathophysiology and emphasize the need for a comprehensive approach to studying this phenomenon.

This study also sheds light on the potential of salivary oxidative biomarkers, such as glutathione and vitamin E, as indicators of disease presence and progression. However, the hypothesis that antioxidant supplementation could serve as a preventive strategy remains speculative, given that the study did not include interventional analyses or a detailed exploration of confounding variables. Future research should focus on longitudinal designs, incorporating dietary, genetic, and behavioral factors, to better elucidate the dynamic relationship between oxidative stress and dental caries.

The findings of this study must be interpreted within the context of several limitations. First, its cross-sectional nature precludes the establishment of causal relationships between antioxidant levels and dental caries. Second, potential confounding factors, including dietary habits, oral hygiene practices, and genetic predispositions, were not accounted for, limiting the robustness of the conclusions. Third, the reliance on participants from a single geographic location restricts the generalizability of the results. Lastly, the study did not evaluate temporal changes in antioxidant levels or the impact of targeted interventions, which could provide critical insights into the preventive and therapeutic roles of these markers in dental caries.

## Conclusions

This study investigated the association between salivary antioxidant levels, specifically GPx and vitamin E, and the presence of dental caries in children. The findings indicated that children with caries exhibited significantly lower levels of these antioxidants compared to their caries-free counterparts, suggesting a compromised oxidative defense mechanism. These results align with prior research, emphasizing the critical role of antioxidants in protecting oral tissues from oxidative damage caused by ROS, which are implicated in caries pathogenesis. The observed disparity in antioxidant levels highlights the potential for enhancing salivary antioxidant capacity as a preventive or therapeutic strategy for dental caries. However, the cross-sectional nature of this study limits the ability to establish causality. Additionally, confounding factors such as dietary habits, oral hygiene practices, and genetic predispositions, which could influence antioxidant levels and caries susceptibility, were not controlled. Despite these limitations, the findings underscore the importance of maintaining a robust salivary antioxidant defense for optimal oral health. Further longitudinal and interventional studies are required to validate these findings and explore strategies to bolster salivary antioxidant capacity, such as dietary modifications or targeted supplementation. Such approaches may provide novel insights into reducing the prevalence of dental caries through nutritional and biochemical interventions. Ultimately, this study contributes to the growing body of evidence linking oxidative stress to dental caries and highlights the need for continued research to address the multifactorial nature of this widespread oral disease.
